# Short-term longitudinal associations among parent–child relationship quality, internalizing and externalizing problems, and belief in a just world: a three-wave RI-CLPM

**DOI:** 10.3389/fpsyg.2026.1914539

**Published:** 2026-09-01

**Authors:** Linxin Guo, Yayun Wang, Yueyue Zhou

**Affiliations:** 1 School of Continuing Education, Luoyang Vocational College of Culture and Tourism, Luoyang, China; 2 Department of Psychology, Henan University, Kaifeng, China

**Keywords:** adolescent, belief in a just world, internalizing and externalizing problems, parent–child relationship, random intercept cross-lagged model

## Abstract

Parent–child relationship quality is associated with adolescent internalizing and externalizing problems, but conventional cross-lagged models do not separate stable between-person differences from within-person fluctuations. This three-wave study examined short-term reciprocal associations among parent–child relationship quality, belief in a just world (BJW), and internalizing and externalizing problems, as well as indirect effects through BJW and differences by gender. Participants were 591 students from one middle school in Henan Province, China (*M_age_* = 13.30 years, *SD* = 0.94; 51.8% boys). Assessments were conducted at one-month intervals, and the entire assessment period spanned 2 months. Random-intercept cross-lagged panel models indicated that, at the within-person level, better-than-usual parent–child relationship at T1 and T2 predicted stronger subsequent BJW (β_T1-T2_ = 0.08, *p* < 0.05; β_T2-T3_ = 0.11, *p* < 0.05) and fewer subsequent internalizing and externalizing problems (β_T1-T2_ = −0.20, *p* < 0.001; β_T2-T3_ = −0.22, *p* < 0.001). BJW and internalizing and externalizing problems did not predict subsequent parent–child relationship. BJW and internalizing and externalizing problems showed reciprocal negative associations, but neither indirect pathway through BJW was significant. The multi-group comparison indicated gender heterogeneity in the cross-lagged system, although the indirect effects did not differ by gender and only two individual paths differed significantly.

## 1 Introduction

The high prevalence of internalizing and externalizing problems among adolescents, along with their negative impacts, has made them a major topic of psychological research and a focal point of public health (Bista et al., 2025; Bor et al., 2014; Jia et al., 2024). Internalizing and externalizing problems are commonly used as indicators to assess an individual’s mental health. Internalizing and externalizing problems are related but conceptually distinct domains of adolescent maladjustment. Internalizing problems direct distress inward and commonly include anxiety, depressed mood, withdrawal, and somatic complaints; externalizing problems are expressed outwardly through aggression, rule-breaking, and other dysregulated behavior(Buchanan-Pascall et al., 2018; Achenbach et al., 2016). The two domains often co-occur, but they may arise through partly different developmental processes and carry different interpersonal consequences (Bista et al., 2025; Buchanan-Pascall et al., 2018). Persistent difficulties in either domain are associated with impaired social functioning and elevated risk of later maladjustment (Dubowitz et al., 2021). Identifying modifiable relational and cognitive correlates is therefore important for prevention, while retaining the conceptual distinction between emotional distress and behavior directed toward others. Previous research indicates that the parent–child relationship as a key interpersonal resource for adolescents and an important factor in the understanding of the development of their internalizing and externalizing problems (Barger et al., 2019; Rothenberg et al., 2020; Shi et al., 2022). At the same time, the belief in a just world, as a key component of an individual’s inner resources, is an important factor in ensuring mental well-being (Cao et al., 2021; Weinberg et al., 2019). A healthy parent–child relationship may indirectly influence the occurrence of internalizing and externalizing problems by fostering adolescents’ belief in a just world (Correia and Dalbert, 2008; Donat et al., 2018). However, existing research has failed to distinguish between within-individual and between-individual effects. The direction of the relationship between parent–child relationships, belief in a just world, and internalizing and externalizing problems among adolescents remains unclear at the individual level. Therefore, this study aims to use a random intercept cross-lagged model (RI-CLPM) to examine the longitudinal relationships among parent–child relationships, belief in a just world, and internalizing and externalizing problems.

### 1.1 Parent–child relationship quality and adolescent adjustment

Parent–child relationship quality refers here to adolescents’ perceived emotional closeness, cohesion, support, and positive interaction with their parents. During adolescence, this relationship remains a central context for emotion regulation, coping, and behavioral guidance even as autonomy and peer involvement increase (Collins and Laursen, 2004; Shi et al., 2022). Warm and responsive relationships may provide emotional security, make parental guidance easier to accept, and support constructive coping (Jiang et al., 2023; Liu et al., 2019). In contrast, conflict, rejection, and psychological control may heighten threat sensitivity, undermine regulation, and increase both emotional distress and acting-out behavior (Giannotta and Rydell, 2016; McKinney et al., 2016; Murray et al., 2014; Xiao et al., 2017; Zou et al., 2022).

The transactional model of development proposes that children and their relational environments influence one another over time (Sameroff and Mackenzie, 2003). Applied to adolescence, this model supports two directional expectations. First, a deterioration in parent–child relationship quality may precede increases in internalizing and externalizing problems because adolescents receive less support and experience more interpersonal stress. Second, emotional and behavioral problems may alter parent-adolescent interactions by increasing conflict, eliciting stricter control, or reducing constructive communication (Zhang R. et al., 2024; Zhang Y. et al., 2024). Evidence of either pathway should nevertheless be interpreted as temporal prediction rather than proof of causation, because cross-lagged observational data cannot rule out unmeasured common causes.

The meaning of parent–child interaction is also culturally situated. Chinese family relationships may combine parental authority, involvement, obligation, and practices understood as training, and these features are not always captured adequately by a simple Western authoritarian-authoritative contrast (Chao, 1994). Adolescents’ interpretations of parental behavior may therefore depend on whether guidance is experienced as care, legitimate authority, psychological control, or unfair treatment. This context may shape both perceived relationship quality and the extent to which family experiences are generalized into beliefs about fairness. However, it must be acknowledged that Chinese families exhibit significant heterogeneity in terms of regional distribution and socioeconomic backgrounds, and therefore culture should not be treated as a uniform explanatory factor.

### 1.2 Belief in a just world as a proposed mechanism

Belief in a just world is rooted in Lerner’s just-world theory rather than social cognitive theory. It refers to the tendency to perceive the world as orderly and to expect that people generally receive outcomes they deserve (Lerner, 1980; Lerner and Miller, 1978). Personal BJW concerns fairness in outcomes for oneself, whereas general BJW concerns fairness in the wider social world (Dalbert, 1999). BJW can function as a psychological resource by supporting perceived predictability, meaning, future orientation, and coping, although rigid just-world beliefs may also have maladaptive implications in some contexts (Bartholomaeus and Strelan, 2019; Hafer et al., 2020; Lutz et al., 2025).

There is a specific, although not exclusive, rationale for examining BJW as a link between parent–child relationships and adjustment. Family interactions are among adolescents’ most frequent experiences of authority, rules, reward, punishment, and interpersonal treatment. Consistent, respectful, and supportive treatment may provide repeated evidence that important social relationships are predictable and fair. These experiences may be generalized into stronger expectations of personal and social justice (Correia and Dalbert, 2008; Feng et al., 2024). Stronger BJW may then reduce distress and dysregulated behavior by helping adolescents interpret setbacks as manageable, maintain future-oriented goals, and use less hostile or helpless appraisals (Liu and Platow, 2020; Weinberg et al., 2023; Zhou et al., 2025). Empirical research has demonstrated that the belief in a just world positively predicts an individual’s mental health (Hafer et al., 2020), interpersonal relationships (Sutton et al., 2017), and prosocial behavior (Guo et al., 2022). This proposed pathway distinguishes BJW from broader constructs such as attachment security, self-esteem, and interpersonal trust, but it does not imply that BJW is the only or necessarily the strongest indirect factor.

The alternative common-cause interpretation also remains plausible. A stable supportive family environment could independently promote BJW and reduce problem behaviors without BJW carrying the effect of parent–child relationship quality. Longitudinal temporal ordering can test whether earlier relationship fluctuations precede later BJW and whether BJW precedes later problems, but it cannot by itself establish the complete causal mechanism. Moreover, prior studies have mostly examined pairwise associations among parenting, BJW, and adjustment, without directly examined the prospective indirect effects of BJW.

Reciprocal pathways involving BJW are also possible. Internalizing symptoms can bias interpretations toward hopelessness and unfairness, whereas externalizing behavior can elicit rejection and punishment that reinforce perceptions of an unjust social world (Beck, 2008; Glenn et al., 2021; Rubenstein et al., 2016; Sun et al., 2024; Wright et al., 2020). BJW may therefore predict later adjustment, and adjustment difficulties may in turn erode BJW. A reverse indirect pathway from problem behaviors to parent–child relationship quality through BJW is theoretically less established than the forward pathway and is treated as exploratory in this study.

### 1.3 Gender, socialization, and relational sensitivity

Gender schema theory was introduced by Bem (1981) and proposes that culturally learned gender schemas guide attention, self-concept, and behavior. In family and peer contexts, gender socialization may influence acceptable emotional expression, help-seeking, independence, and responses to interpersonal strain. Girls often report greater interpersonal orientation and may be more sensitive to relationship stress, whereas boys may face stronger norms favoring emotional restraint and behavioral expressions of distress (Chaplin and Aldao, 2013; Rose and Rudolph, 2006; Schleider et al., 2014). These tendencies are probabilistic rather than universal and may be shaped by culture, family practice, and developmental stage.

Prior evidence does not establish a consistent gender pattern for all links among parent–child relationships, BJW, and adjustment. We therefore distinguish a theory-driven primary expectation from more tentative comparisons. Based on the interpersonal-sensitivity account, we expected the prospective association between poorer parent–child relationship quality and greater adjustment problems, and the corresponding indirect association through BJW, to be stronger among girls. Other gender differences in BJW-related paths were examined as secondary comparisons.

### 1.4 The current study

Traditional cross-lagged panel models may conflate stable between-person differences with within-person fluctuations. The random-intercept cross-lagged panel model (RI-CLPM) separates adolescents’ stable average levels from time-specific deviations, permitting a more focused test of whether being above or below one’s usual level on one construct predicts subsequent deviations in another. The present three-wave study used RI-CLPM to examine short-term associations among parent–child relationship quality, BJW, and internalizing and externalizing problems.

We tested the following hypotheses. H1a predicted that better-than-usual parent–child relationship quality would predict fewer subsequent internalizing and externalizing problems. H1b predicted that greater-than-usual problems would predict poorer subsequent parent–child relationship quality, consistent with a transactional account. H2a predicted that better parent–child relationship quality would predict stronger subsequent BJW, and H2b predicted that stronger BJW would predict fewer subsequent problems. H2c predicted a prospective indirect pathway from parent–child relationship quality to later problems through BJW. Reverse pathways from problems to parent–child relationship quality through BJW were exploratory because their theoretical basis is weaker. H3 predicted that the bidirectional indirect effects of belief in a just world between parent–child relationship quality and internalizing and externalizing problems would differ by gender, and the effects would be stronger for girls than for boys.

## 2 Method

### 2.1 Participants

This study employed convenience sampling to select students from 12 classes in grades 7 through 9 at a junior high school in Xuchang, Henan Province, China, as the survey participants. A total of 700 questionnaires were distributed. At the first assessment (T1), 647 questionnaires were actually returned; at the second (T2) and third (T3) assessments, 595 and 595 were returned, respectively. Data collection began in mid-October 2023, with three assessments spaced 1 month apart, covering an observation period of about 2 months. Some students were unable to complete all assessments due to objective reasons such as taking leave. After data matching and quality review, and after excluding repetitive responses and invalid questionnaires, a valid sample of 591 students who fully participated in all three assessments was ultimately included. The participant attrition rate was 8.70% (56/647), and the response rate was 84.43% (591/700). This included 306 boys (51.8%) and 285 girls (48.2%). Participants were 12 to 16 years old (*M_age_* = 13.30 years, *SD* = 0.94).

### 2.2 Measures

#### 2.2.1 Parent–child relationship quality

Adolescents’ perceived parent–child relationship quality was assessed with the Chinese parent–child cohesion measure described by Wang and Zhang (2007), adapted from the cohesion dimension of the Family Adaptability and Cohesion Evaluation Scales. The same 10 prompts were rated separately for relationships with fathers and mothers on a scale from 1 (almost never) to 5 (almost always). The primary analysis calculated the total scores for father-child and mother–child scores to represent overall perceived parent–child relationship quality, with higher total scores indicating better relationship quality. Cronbach’s alpha was 0.86, 0.88, and 0.83 across the three waves.

#### 2.2.2 Internalizing and externalizing problems

The revised Chinese version of the Achenbach Youth Self-Report (YSR-2001-CV; Wang et al., 2013) was used to assess adolescent problems. The instrument contains 79 items covering aggression, rule-breaking, anxiety/depression, withdrawal, social problems, somatic complaints, attention problems, and thought problems. Aggression and rule-breaking were used as indicators of externalizing problems, whereas anxiety/depression, somatic complaints, and withdrawal were used as indicators of internalizing problems. Items were rated from 0 (neve) to 2 (often). The primary analysis calculated the selected items into a single composite problem score, with higher total score indicating more problems. Cronbach’s alpha was 0.93, 0.94, and 0.93 across the three waves.

#### 2.2.3 Belief in a just world

Belief in a just world was assessed with Dalbert’s (1999) 13-item scale, which includes personal and general belief in a just world dimension. Items were rated from 1 (strongly disagree) to 6 (strongly agree). The primary analysis calculated the total scores for belief in a just world, rather than estimated personal and general belief in a just world separately, with higher total scores indicating stronger belief in a just world. Cronbach’s alpha was 0.93, 0.94, and 0.91 across the three waves.

### 2.3 Research procedure

Data were collected by trained graduate students in applied psychology during class self-study periods. Administrators followed a standardized script that introduced the study, explained questionnaire completion, and included a confidentiality statement. To comply with legal and ethical standards, all participants signed informed consent forms and were explicitly informed of their right to withdraw from the study at any point. The participants then completed the questionnaire independently, which took approximately 15 to 20 min. Questionnaires were collected immediately, all questionnaires will be treated with strict confidentiality, and the data will be used exclusively by the research team. After completing the questionnaire survey, each participant received a small token of appreciation for their participation.

### 2.4 Statistical analysis

Descriptive statistics and person correlations were calculated in SPSS 19.0. Longitudinal measurement models and random intercept cross-lag model (RI-CLPM) were estimated in Mplus 8.0. Configural, metric, and scalar measurement models were compared across the three waves. Evaluation considered both absolute fit and changes in comparative fit index (CFI); a small change in CFI cannot compensate for poor absolute fit. The primary RI-CLPM separated stable between-person differences from within-person deviations and included autoregressive and cross-lagged paths among parent–child relationship quality, BJW, and the composite problem score. To address the issue of missing data, this study employs the Full Information Maximum Likelihood (FIML) method for handling missing data. Maximum likelihood with robust standard errors (i.e., MLR) was conducted to account for non-normality. Model fit was evaluated with the chi-square statistic, CFI and Tucker-Lewis index (TLI), root mean square error of approximation (RMSEA), and standardized root mean square residual (SRMR). The statistical significance of the indirect effects was tested using the Delta method (i.e., the Sobel test) combined with MLR (maximum likelihood robust) standard errors. Accurate parameter estimates and standard errors are provided by this method given a sufficient sample size (MacKinnon et al., 2002). To examine whether the sample size was adequate, we conducted a Monte Carlo simulation with 1,000 replications (Muthén and Muthén, 2002). Results showed that the statistical power for the indirect effect in the full sample (*N* = 591) was 0.966, well above the recommended benchmark of 0.80 (Cohen, 1988), indicating that the sample size of this study was sufficient to detect the hypothesized indirect effect.

To examine whether the associations differed significantly by gender, multi-group analyses were conducted. An unconstrained baseline model (with all cross-lagged paths freely estimated across male and female groups) was compared with a constrained model in which all cross-lagged path coefficients were set equal across groups. A non-significant deterioration in model fit (i.e., a non-significant Satorra-Bentler corrected Δχ^2^, and ΔCFI ≤ 0.01; Cheung and Rensvold, 2002) would indicate that the dynamic relationships between variables were invariant across male and female groups.

## 3 Results

### 3.1 Descriptive statistics and correlations

**Figure 1:**
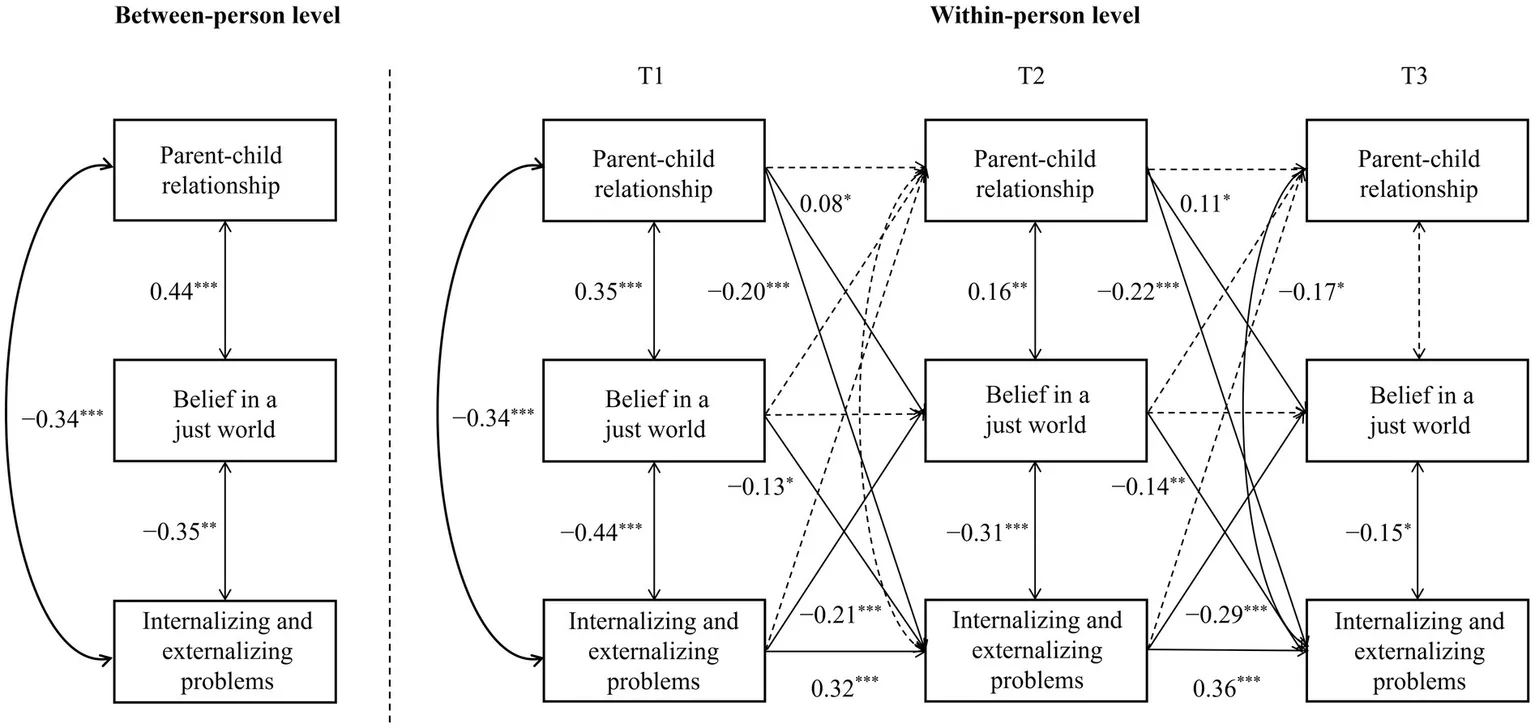
Random-intercept cross-lagged panel model of parent–child relationship quality, belief in a just world, and internalizing and externalizing problems. Coefficients are standardized. Solid lines indicate statistically significant paths; dashed lines indicate nonsignificant paths. T1 = Time 1; T2 = Time 2; T3 = Time 3.^*^p < 0.05, ^**^p < 0.01, ^***^p < 0.001.

Descriptive statistics and correlations are presented in Table 1. At each wave, parent–child relationship quality was negatively correlated with internalizing and externalizing problems (*r* = −0.19 to −0.37, *p* < 0.001) and positively correlated with BJW (*r* = 0.25 to 0.38, *p* < 0.001). BJW was negatively correlated with problems across waves (*r* = −0.21 to −0.45, *p* < 0.001). Table 2 presents gender comparisons. Girls reported higher composite problem scores than boys at all three waves (t_T1_ = −4.04, *p* < 0.01; t_T2_ = −3.02, *p* < 0.01; t_T3_ = −3.35, *p* < 0.001). The other mean gender differences were not statistically significant (see Figure 1).

**Table 1:** Descriptive statistics and correlations among the study variables.

Variable	1	2	3	4	5	6	7	8	9	10
1. Gender	—									
2. T1 Internalizing and externalizing problems	0.16^**^	—								
3. T2 Internalizing and externalizing problems	0.12^**^	0.66^***^	—							
4. T3 Internalizing and externalizing problems	0.14^**^	0.53^***^	0.63^***^	—						
5. T1 Parent–child relationship	−0.07	−0.35^***^	−0.37^***^	−0.28^***^	—					
6. T2 Parent–child relationship	−0.06	−0.25^***^	−0.24^***^	−0.32^***^	0.58^***^	—				
7. T3 Parent–child relationship	0.02	−0.19^***^	−0.22^***^	−0.27^***^	0.55^***^	0.56^***^	—			
8. T1 Belief in a just world	−0.05	−0.42^***^	−0.45^***^	−0.28^***^	0.38^***^	0.27^***^	0.25^***^	—		
9. T2 Belief in a just world	0.02	−0.32^***^	−0.42^***^	−0.27^***^	0.28^***^	0.31^***^	0.28^***^	0.53^***^	—	
10. T3 Belief in a just world	−0.01	−0.21^***^	−0.32^***^	−0.27^***^	0.27^***^	0.28^***^	0.30^***^	0.49^***^	0.44^***^	—
*M*		0.64	0.60	0.57	1.74	1.74	1.72	3.59	3.17	3.77
*SD*		0.23	0.25	0.23	0.32	0.33	0.29	1.12	1.17	0.94

^**^p < 0.01; ^***^p < 0.001. T1 = Time 1; T2 = Time 2; T3 = Time 3.

**Table 2:** Gender differences in the study variables across waves.

Variable	Boy (*n* = 306)	Girl (*n* = 285)	Total (*n* = 591)	*t*
	Mean (SD)	Mean (SD)	Mean (SD)	
T1 Internalizing and externalizing problems	50.87 (18.09)	57.14(19.72)	53.89 (19.14)	−4.04^***^
T2 Internalizing and externalizing problems	48.35 (19.92)	53.44 (21.06)	50.80 (20.62)	−3.02^**^
T3 Internalizing and externalizing problems	45.43 (19.54)	50.79 (19.26)	48.01 (19.57)	−3.35^***^
T1 Parent–child relationship	35.29 (6.44)	34.39 (6.15)	34.85 (6.31)	1.74
T2 Parent–child relationship	35.24 (6.59)	34.38 (6.63)	34.82 (6.62)	1.57
T3 Parent–child relationship	34.32 (5.86)	34.49 (5.73)	34.40 (5.79)	−0.38
T1 Belief in a just world	47.35 (14.72)	45.98 (14.36)	46.69 (14.55)	1.15
T2 Belief in a just world	41.00 (15.20)	41.53 (15.29)	41.25 (15.23)	−0.43
T3 Belief in a just world	49.12 (12.44)	48.94 (12.06)	49.04 (12.25)	0.18

^**^p < 0.01; ^***^p < 0.001. T1 = Time 1; T2 = Time 2; T3 = Time 3.

### 3.2 Longitudinal measurement evaluation

**Figure 2:**
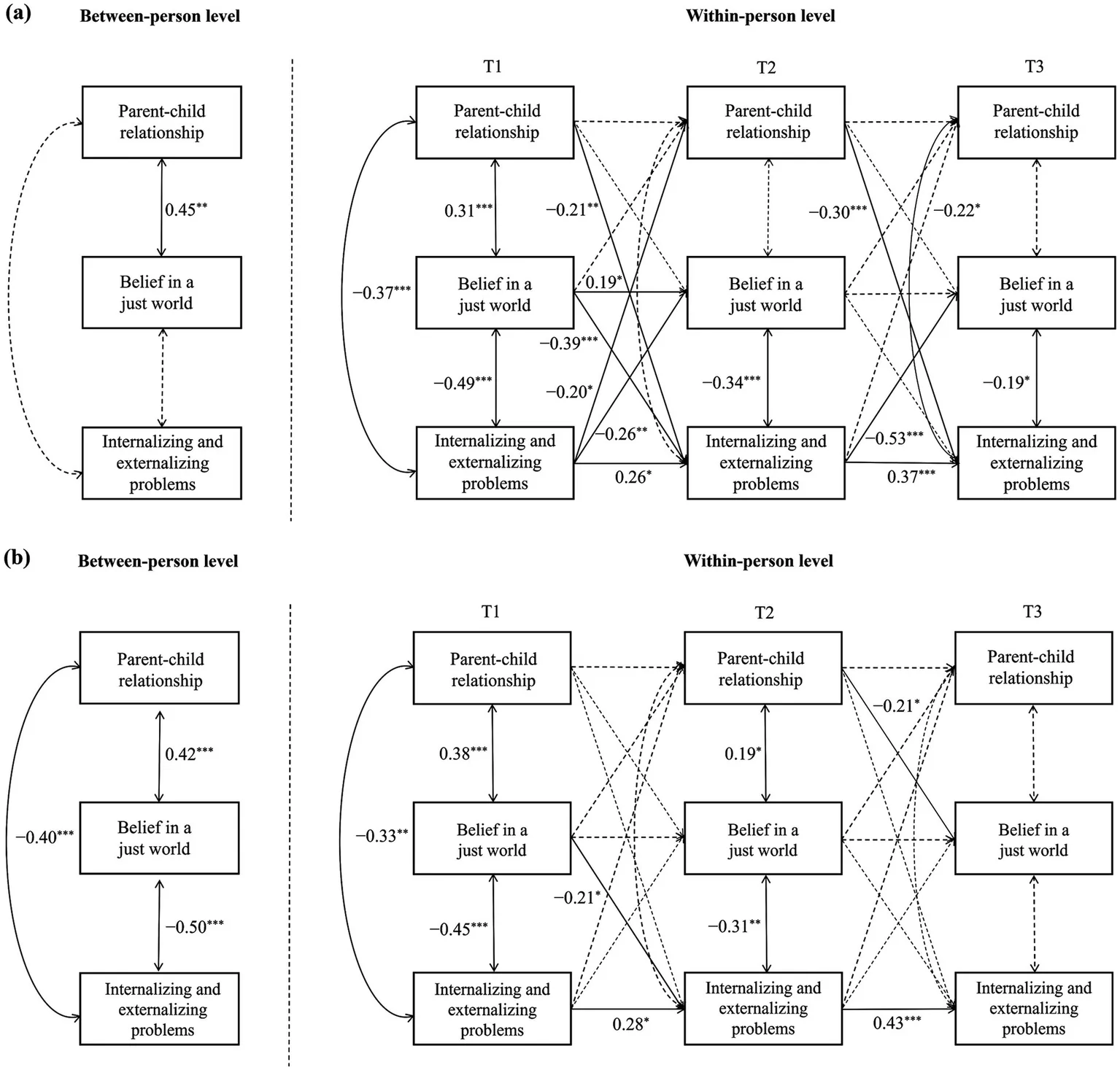
Gender multi-group random-intercept cross-lagged panel models of parent–child relationship quality, belief in a just world, and internalizing and externalizing problems. **(a)** = boys; **(b)** = girls. Coefficients are standardized. Solid lines indicate statistically significant paths; dashed lines indicate nonsignificant paths. *T1* = Time 1; *T2* = Time 2; *T3* = Time 3. ^*^p < 0.05, ^**^p < 0.01, ^***^p < 0.001.

This study employed confirmatory factor analysis (CFA) to examine the longitudinal measurement invariance of the parent–child relationships, the belief in a just world, and adolescents’ internalizing and externalizing problems across three measurement waves. Following the criteria of Chen (2007) and Cheung and Rensvold (2002), we adopted ΔCFI ≤ |0.01| and ΔRMSEA ≤ 0.015 as cutoff criteria for invariance. The results are presented in Table 3. The internalizing and externalizing problem scales both reached scalar invariance, suggesting that factor loadings and intercepts were invariant across the three waves, thereby meeting the assumption for longitudinal latent variable modeling. In contrast, the belief in a just world and parent–child relationship scales achieved only metric invariance (ΔCFI = −0.010), but failed to reach scalar invariance (ΔCFI = −0.068 and −0.035, respectively). As Putnick and Bornstein (2016) noted, metric invariance represents the minimum prerequisite for comparing structural parameters across time (e.g., cross-lagged paths). Accordingly, although not all variables reached scalar invariance, the within-person cross-lagged effects for the key variables in this study were considered comparable over time (see Figure 2).

**Table 3:** Longitudinal measurement-model evaluation for the study variables.

Measuring tools	Model	χ^2^ (df)	ΔCFI	CFI	TLI	RMSEA (90% CI)	SRMR	ΔRMSEA
Internalizing problems	M1	4049.172 (2205)		0.850	0.841	0.038 [0.036–0.039]	0.049	
	M2	4102.761 (2249)	0.000	0.850	0.843	0.037 [0.036–0.039]	0.051	0.002
	M3	4227.213 (2291)	−0.007	0.843	0.839	0.038 [0.036–0.040]	0.052	0.001
Externalizing problems	M1	5566.787 (3075)		0.788	0.776	0.037 [0.035–0.039]	0.054	
	M2	5596.638 (3127)	0.001	0.789	0.782	0.037 [0.035–0.038]	0.056	0.002
	M3	5762.662 (3177)	−0.009	0.780	0.775	0.037 [0.036–0.039]	0.056	0.000
Parent–child relationship	M1	10314.296 (1647)		0.826	0.813	0.094 [0.093–0.096]	0.098	
	M2	10851.055 (1667)	−0.010	0.816	0.805	0.097 [0.095–0.098]	0.111	0.010
	M3	12729.959 (1765)	−0.035	0.781	0.773	0.103 [0.101–0.104]	0.155	0.044
Belief in a just world	M1	2522.848 (660)		0.865	0.849	0.069 [0.066–0.072]	0.055	
	M2	2672.247 (672)	−0.010	0.855	0.840	0.071 [0.068–0.074]	0.067	0.006
	M3	3964.137 (826)	−0.068	0.787	0.778	0.080 [0.078–0.083]	0.092	0.008

M1 = configural invariance; M2 = metric invariance; M3 = scalar invariance; CFI stands for the Comparative Fit Index; TLI stands for the Tucker-Lewis Index; RMSEA stands for the Root Mean Square Error of Approximation; SRMR stands for the Standardized Root Mean Square Residual.

### 3.3 Random-intercept cross-lagged panel model

The three-wave RI-CLPM showed acceptable overall fit, χ^2^ (12) = 44.44, CFI = 0.98, TLI = 0.94, SRMR = 0.05, RMSEA = 0.07. At the between-person level, adolescents with higher average BJW reported fewer average problems (*r* = −0.35, *p* < 0.01 Figure 1). Higher average parent–child relationship quality was associated with fewer average problems (*r* = −0.34, *p* < 0.001) and stronger average BJW (*r* = 0.44, *p* < 0.001). These between-person correlations do not indicate temporal direction.

At the within-person level, parent–child relationship quality predicted subsequent deviations in both BJW and problems. Better-than-usual relationship quality predicted stronger subsequent BJW (β_T1–T2_ = 0.08, *p* < 0.05; β_T2-T3_ = 0.11, *p* < 0.05) and fewer subsequent problems (β_T1–T2_ = −0.20, *p* < 0.001; β_T2–T3_ = −0.22, *p* < 0.001). Neither BJW nor problems significantly predicted subsequent parent–child relationship quality. BJW and problems showed reciprocal negative paths. Stronger-than-usual BJW predicted fewer subsequent problems (β_T1–T2_ = −0.13, *p* < 0.05; β_T2–T3_ = −0.14, *p* < 0.01), and greater-than-usual problems predicted weaker subsequent BJW (β_T1–T2_ = −0.21, *p* < 0.001; β_T2–T3_ = −0.29, *p* < 0.001).

### 3.4 The prospective indirect effects of belief in a just world

Table 4 presents the prospective indirect effects. The pathway from T1 parent–child relationship quality to T3 problems through T2 BJW was not significant (*β* = −0.01, *p* = 0.115, 95% CI [−0.025, 0.003]). The reverse pathway from T1 problems to T3 parent–child relationship quality through T2 BJW was also not significant (*β* = −0.02, *p* = 0.171, 95% CI [−0.048, 0.008]).

**Table 4:** Prospective indirect effects.

Path	*β*	*p*	95% CI	
			Boot LL	Boot UL
T1 Parent–child relationship → T2 Belief in a just world → T3 Internalizing and externalizing problems	−0.01	0.115	−0.025	0.003
T1 Internalizing and externalizing problems → T2 Belief in a just world → T3 Parent–child relationship	−0.02	0.171	−0.048	0.008

### 3.5 Multiple-group analysis

See Table 5, the scaled chi-square difference test comparing the freely estimated and equality-constrained multigroup models was significant, ΔS-Bχ^2^(27) = 56.73, *p* < 0.001, indicating that at least one cross-lagged coefficient differed between boys and girls. Follow-up Wald tests identified two path differences. Parent–child relationship quality at T2 predicted fewer problems at T3 among boys (*β* = −0.30, *p* < 0.001) but not among girls (*β* = −0.11, *p* = 0.209). Problems at T2 predicted weaker BJW at T3 among boys (*β* = −0.53, *p* < 0.001) but not among girls (*β* = −0.16, *p* = 0.179). The prospective indirect effects through BJW were not significant in either gender group. For the relationship-to-problems pathway, the 95% confidence intervals were [−0.01, 0.01] for boys and [−0.07, 0.06] for girls. For the reverse pathway, the intervals were [−0.10, 0.06] for boys and [−0.03, 0.01] for girls (see Figure 2).

**Table 5:** Model comparisons for the gender multigroup random-intercept cross-lagged panel models.

Model	χ^2^	df	Scaling factor	RMSEA	CFI	TLI	SRMR	ΔS-Bχ^2^	Δdf	Comparison model	*p*
M1	8.996	6	0.923	0.041	0.998	0.978	0.015				
M2	65.792	33	1.046	0.058	0.980	0.956	0.053	56.73	27	M1 vs. M2	<0.001

M1, freely estimated across gender groups; M2, cross-lagged paths constrained to equality across gender groups.

## 4 Discussion

This study used a three-wave RI-CLPM to separate stable between-person differences from short-term within-person fluctuations in parent–child relationship quality, BJW, and adolescent problems. Four findings were central. First, better-than-usual parent–child relationship quality predicted fewer problems 1 month later, but problems did not predict subsequent relationship quality. Second, better relationship quality predicted stronger subsequent BJW. Then, although BJW and problem behaviors exhibited negative bidirectional temporal associations over time, the prospective indirect effect of BJW between parent–child relationship quality and adolescent problem behaviors was not significant. Finally, the omnibus gender comparison was significant, although the hypothesized stronger relationship effects among girls were not supported and only two individual paths differed. These findings provide new evidence for understanding the influence of parent–child relationships on adolescents’ internalizing and externalizing problems.

### 4.1 Parent–child relationship quality and adolescent problems

This study found that the parent–child relationship at time points T1 and T2 negatively predicted the internalizing and externalizing problems of adolescents at time points T2 and T3, respectively. This means that when adolescents perceive their parent–child relationship quality as better than their usual level, their subsequent problems decrease correspondingly. A positive parent–child relationship provides adolescents with emotional security and a sense of belonging, enabling them to receive timely emotional support and guidance on coping strategies when facing stress and challenges, thereby reducing internalizing and externalizing problems caused by emotional distress and behavioral issues (Tang et al., 2025). In addition, a positive parent–child relationships also help adolescents cultivate positive psychological qualities, enabling them to adopt proactive coping strategies when facing problems, which in turn reduces the occurrence of internalizing and externalizing problems (Huang et al., 2019; Wang and Lin, 2018). Conversely, if adolescents are exposed to an insecure parent–child relationship environment over an extended period, they are more likely to develop negative emotions and exhibit more internalizing and externalizing problems (Zhao et al., 2024). However, potential variables such as emotional security and positive psychological qualities were not directly measured in this study, so the above explanations await further validation. Future research could address this limitation by incorporating these key variables to provide a more comprehensive understanding of the mechanisms through which parent–child relationship quality shapes adolescent psychological adjustment.

In addition, this study found that adolescents’ internalizing and externalizing problems did not reverse predict their parent–child relationship quality, which is inconsistent with the hypothesis. However, this result does not necessarily imply that adolescents’ problem behaviors are unrelated to the parent–child relationship. One plausible explanation is that changes in parent–child relationship quality may occur more slowly than fluctuations in self-reported emotional and behavioral states, and the one-month interval may not be sufficient to capture corresponding changes in the relationship. Moreover, the aggregation of mother–child and father-child relationships into a single overall indicator may have masked the differential effects of problem behaviors on each parent. Future research could track changes in parent–child interactions over longer time intervals and examine mother–child and father-child relationships separately to more comprehensively elucidate the dynamic associations between adolescent problem behaviors and the parent–child relationship.

### 4.2 The prospective indirect effects of belief in a just world

This study found that parent–child relationship quality at T1 and T2 positively predicted belief in a just world (BJW) at T2 and T3, respectively. This pattern is consistent with a socialization account, in which repeated experiences of being respected, treated predictably, and fairly in core relationships contribute to broader expectations of justice (Correia and Dalbert, 2008; Dalbert, 2001). Moreover, the study revealed significant bidirectional predictive relationships between BJW and adolescents’ internalizing and externalizing problems. Specifically, adolescents with higher levels of BJW were more likely to perceive outcomes as controllable, thereby adopting proactive coping strategies that effectively alleviated internalizing problems such as anxiety and depression, and reduced externalizing behaviors (Hafer and Bègue, 2005; Liu et al., 2020; Song et al., 2018). Conversely, persistent emotional and behavioral difficulties may erode individuals’ basic trust in a just world (Tian et al., 2019). In sum, stronger BJW may sustain a sense of meaning, control, and constructive coping, whereas emotional and behavioral difficulties may increase exposure to negative feedback and bias interpretations toward threat or unfairness (Beck, 2008; Liu and Platow, 2020).

Furthermore, we found no significant indirect effects of BJW between parent–child relationship quality and internalizing/externalizing problems, in either direction. One possible explanation is that BJW, as a relatively stable cognitive belief, may change rather slowly, and the two-month, three-wave design of this study may not have been sufficient to capture this process. In addition, family experiences may influence adolescent adjustment through both fast-acting mechanisms (e.g., immediate emotion regulation) and slow-acting mechanisms (e.g., worldview shaping). The present study focused only on the latter without measuring the former, which may have underestimated the indirect pathway. Future research could extend the follow-up period to capture the long-term developmental effects of BJW, while also incorporating intensive measurement designs (e.g., experience sampling methods) to capture changes in rapid mechanisms such as emotion regulation over short timescales, thereby providing a more comprehensive picture of the multiple temporal pathways through which parent–child relationship quality influences adolescent adjustment.

### 4.3 Gender findings

The results revealed certain gender differences in the cross-lagged relationships among parent–child relationship quality, internalizing and externalizing problems, and belief in a just world. Specifically, the negative predictive path from parent–child relationship to internalizing and externalizing problems, as well as the negative predictive path from internalizing and externalizing problems to belief in a just world, were both significant for boys but not for girls. One possible explanation is that these differences may be related to gender-specific socialization pressures. According to the socialization pressure model, societal expectations encourage male adolescents to be more emotionally restrained and independent (Chaplin and Aldao, 2013). Thus, when parent–child relationship quality declines, boys may be more prone to internalizing or externalizing problems due to a lack of effective emotional expression channels. In contrast, girls may rely on denser social support networks to buffer the adverse effects of the parent–child relationship (Rose and Rudolph, 2006; Zhang R. et al., 2024; Zhang Y. et al., 2024), thereby attenuating the predictive effect of parent–child relationship on problem behaviors. Similarly, male adolescents may be more inclined to attribute negative experiences to external injustice, whereas females may be more likely to attribute them to personal or interpersonal factors (Rose and Rudolph, 2006), which could explain why the predictive effect of problem behaviors on belief in a just world was only significant for boys. However, the present study did not directly measure mechanisms such as emotional suppression, social support, or attribution styles, and the validity of these interpretations awaits future research that directly measures these variables. Therefore, the current findings should be regarded as exploratory evidence of gender differences rather than definitive conclusions.

### 4.4 Cultural context and generalizability

This study was conducted within the Chinese cultural context, where cultural norms may profoundly shape parenting practices, family relationships, and adolescents’ perceptions of justice. It is therefore necessary to discuss the potential role of cultural factors in interpreting the findings. The Chinese context is relevant to how adolescents understand parental authority, family obligations, support, and fairness. Practices experienced as committed guidance or “training” (guan/jiao) in one family may be experienced as intrusive control in another (Chao, 1994). These meanings may simultaneously affect both the reported relationship quality and the generalization of family experiences into belief in a just world. The current measure captures adolescents’ perceptions, which is valuable, but it does not identify the specific parenting practices or cultural meanings underlying those perceptions. Moreover, the sample was drawn from a single middle school in Henan Province and cannot represent the diversity of Chinese adolescents. Variations across regions, urban–rural settings, socioeconomic status, ethnicity, and family structure may alter the observed associations, and cross-cultural generalizability is even more limited. Future research should include multiple regions and schools and should measure culturally meaningful parenting practices, rather than attributing findings broadly to collectivism or Chinese culture.

### 4.5 Theoretical and practical implications

Theoretically, the findings favor an asymmetric short-term pattern: parent–child relationship quality preceded both BJW and problems, while BJW and problems were reciprocally related. This pattern refines a fully symmetric transactional account but does not disprove longer-term child-to-parent effects. It also shows why a plausible pairwise sequence does not guarantee a statistically detectable indirect effect. The time scale of measurement must correspond to the time scale of the proposed psychological process. Moreover, by distinguishing between between-person and within-person effects, this study provides a more nuanced analytical framework for understanding the dynamic associations between parent–child interactions and adolescent psychological adjustment.

Practically, the results suggest that family relationship quality and adolescents’ fairness-related appraisals are relevant assessment domains for school and family mental-health programs. Programs may consider supportive communication, fair and consistent rule-setting, and adolescents’ interpretations of stressful events. However, the observational design does not demonstrate intervention efficacy. Family-based prevention, school counseling, and policy recommendations should therefore be tested experimentally or quasi-experimentally before causal claims are made.

### 4.6 Limitations and future directions

This study has several limitations. First, the sample was drawn from only one middle school in Xuchang, Henan Province, which limits the external validity of the findings. Future research could adopt a cross-regional sampling strategy and include adolescents from diverse socioeconomic and urban–rural backgrounds to enhance the generalizability of the results. Second, the three waves of data were collected at one-month intervals, and this relatively short time window may have limited the ability to detect meaningful changes in relatively stable constructs such as belief in a just world. Future studies should select longer time intervals guided by theory, or combine multiple timescales to capture both slow and fast mechanisms of change. Third, attrition analysis indicated that adolescents with poorer parent–child relationships were more likely to drop out of the study, suggesting possible selection bias that may limit the generalizability of the findings to at-risk populations. Although the attrition rate was relatively low (8.7%), future research should adopt more effective retention strategies to reduce selective attrition and replicate the findings in broader samples. Fourth, all variables in this study were based on adolescent self-reports, which may introduce common method variance, and parental reports of parent–child relationship quality were lacking. Future research could incorporate parent reports or multi-informant assessments to more comprehensively understand the associations between parent–child interactions and adolescent adjustment. Finally, the father-child and mother–child subscales were combined into a total score of parent–child relationship quality, which may have masked the unique influences of fathers and mothers separately, limiting a more nuanced understanding of how each parent contributes to adolescent development. Future research should examine the differential effects of father-child and mother–child relationships separately to enrich dynamic models of parent–child interactions.

## 5 Conclusion

This study examined the longitudinal associations between parent–child relationship quality and adolescents’ internalizing and externalizing problems, and tested the indirect effects of belief in a just world in these associations. Results indicated that, at the within-person level, parent–child relationship quality better than one’s usual level predicted subsequent reductions in adolescent problems and increases in BJW, and that BJW and adolescent problems showed negative reciprocal predictive relationships. However, we did not observe significant prospective indirect effects of BJW between parent–child relationship quality and adolescent problems, nor were there significant gender differences in these indirect effects.

## Data Availability

The raw data supporting the conclusions of this article will be made available by the authors, without undue reservation.
